# Supplementary material for: An NR2F1-dependent retinoic acid network controls retinal specialization in mice and reveals foveal hypoplasia in patients with BBSOAS
Source: bioRxiv. 2026 Sep 22:2026.09.22.753406. Preprint. [Version 1] doi: 10.64898/2026.09.22.753406 (PMC13622096; doi:10.64898/2026.09.22.753406)
Supplement: Supplementary file 1 [file media-1.pdf]

Supplementary Materials for

An NR2F1-dependent retinoic acid network controls retinal specialization in mice and reveals foveal hypoplasia in patients with BBSOAS

Paolo Piovani *et al.*

*Corresponding authors. Email: michele.studer@unice.fr; michele.bertacchi@univ-cotedazur.fr

This PDF file includes:

Figs. S1 to S9
Tables S1 to S5

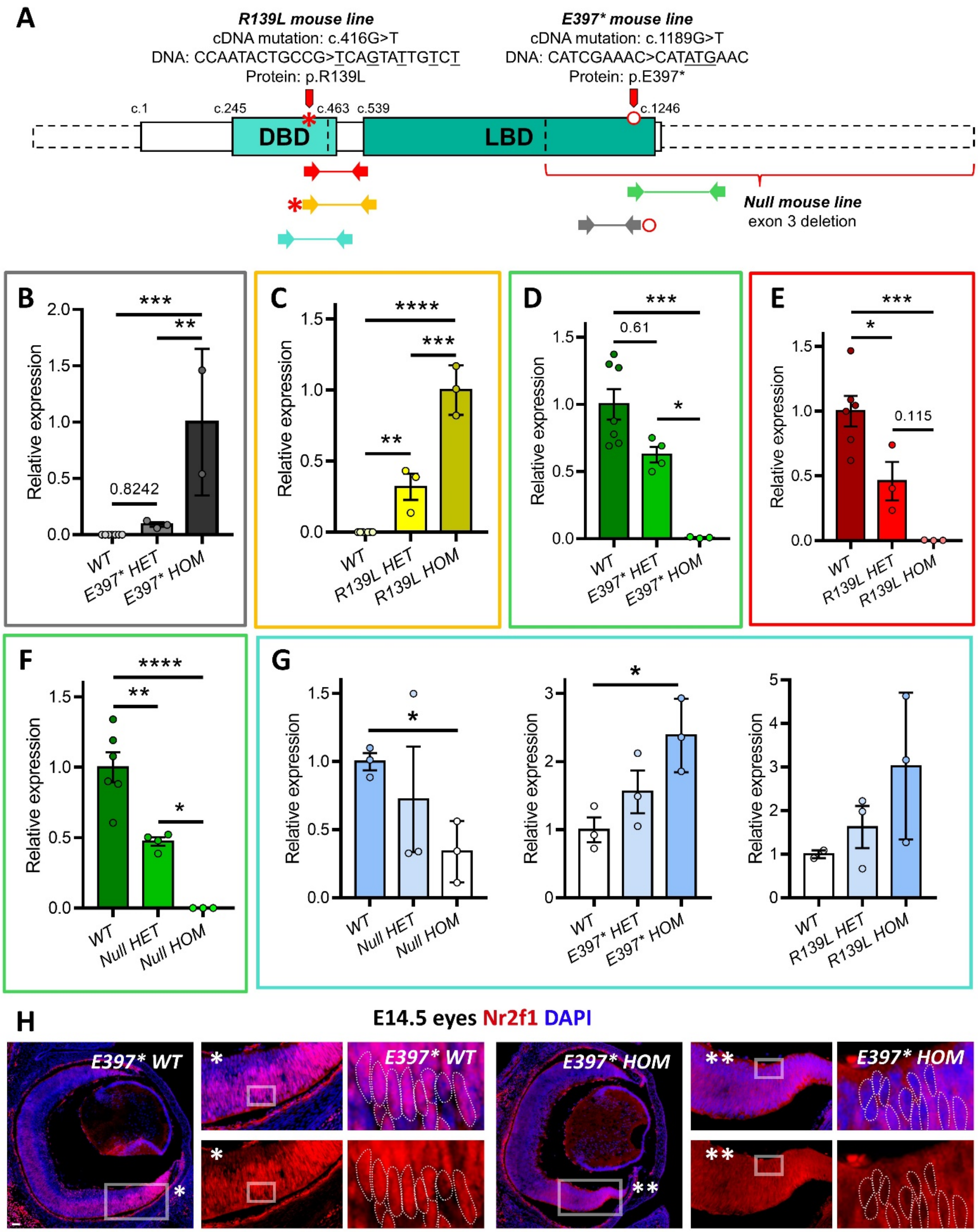

A
R139L mouse line
cDNA mutation: c.416G>T
DNA: CCAATACTGCCG>TCAGTATTGTCT
Protein: p.R139L
E397* mouse line
cDNA mutation: c.1189G>T
DNA: CATCGAAAC>CATATGAAC
Protein: p.E397*
c.1
c.245
c.463
c.539
c.1246
DBD
LBD
Null mouse line
exon 3 deletion
B
C
D
E
F
G
Relative expression
WT
E397* HET
E397* HOM
R139L HET
R139L HOM
Null HET
Null HOM
0.8242
0.61
0.115
H
E14.5 eyes Nr2f1 DAPI
E397* WT
E397* HOM

Fig. S1. Analysis of *Nr2f1* mRNA expression and mutant protein localization. A) Nr2f1 sequence showing the genetic modifications introduced in the three mouse lines and their localization, together with the positions of the primer pairs used to detect *WT* or mutant *Nr2f1* mRNA. The linear protein structure is shown by solid lines superimposed on dotted lines indicating the corresponding mRNA exons. **B–G)** Real-time qRT-PCR quantification of *WT* or mutant *Nr2f1* mRNA expression in embryonic brains from different genotypes, using the same color code as in A to indicate the corresponding primer pairs. Primers specific for the mutant allele detected mutant *Nr2f1* expression in E397* and *R139L HET* and *HOM* embryos (B,C), while WT-specific primers revealed a corresponding reduction in *WT Nr2f1* expression (D,E). Primers targeting exon 3 detected reduced *Nr2f1* expression in *Null HET* and *HOM* embryos (F), whereas universal control primers detected increased total *Nr2f1* mRNA levels in E397* and R139L mutant embryos (G). **H)** Nr2f1 (red) immunostaining in E397* *WT* and *HOM* eyes highlights the cytoplasmic localization of the truncated Nr2f1 protein in the ventral retina. Insets show higher-magnification views highlighting the nuclear localization of Nr2f1 in *E397* WT* eyes (insets marked with *) and its cytoplasmic localization in *E397* HOM* eyes (insets marked with **). Dashed lines in the insets delineate the position of representative RPC nuclei. Scale bars: 50 µm. Each point in graphs B-G corresponds to average expression level in one embryonic brain; n = 2-7 embryos per genotype. Statistical significance was determined by one-way ANOVA with Tukey's correction; * $p < 0.05$; ** $p < 0.01$; *** $p < 0.001$; **** $p < 0.0001$.

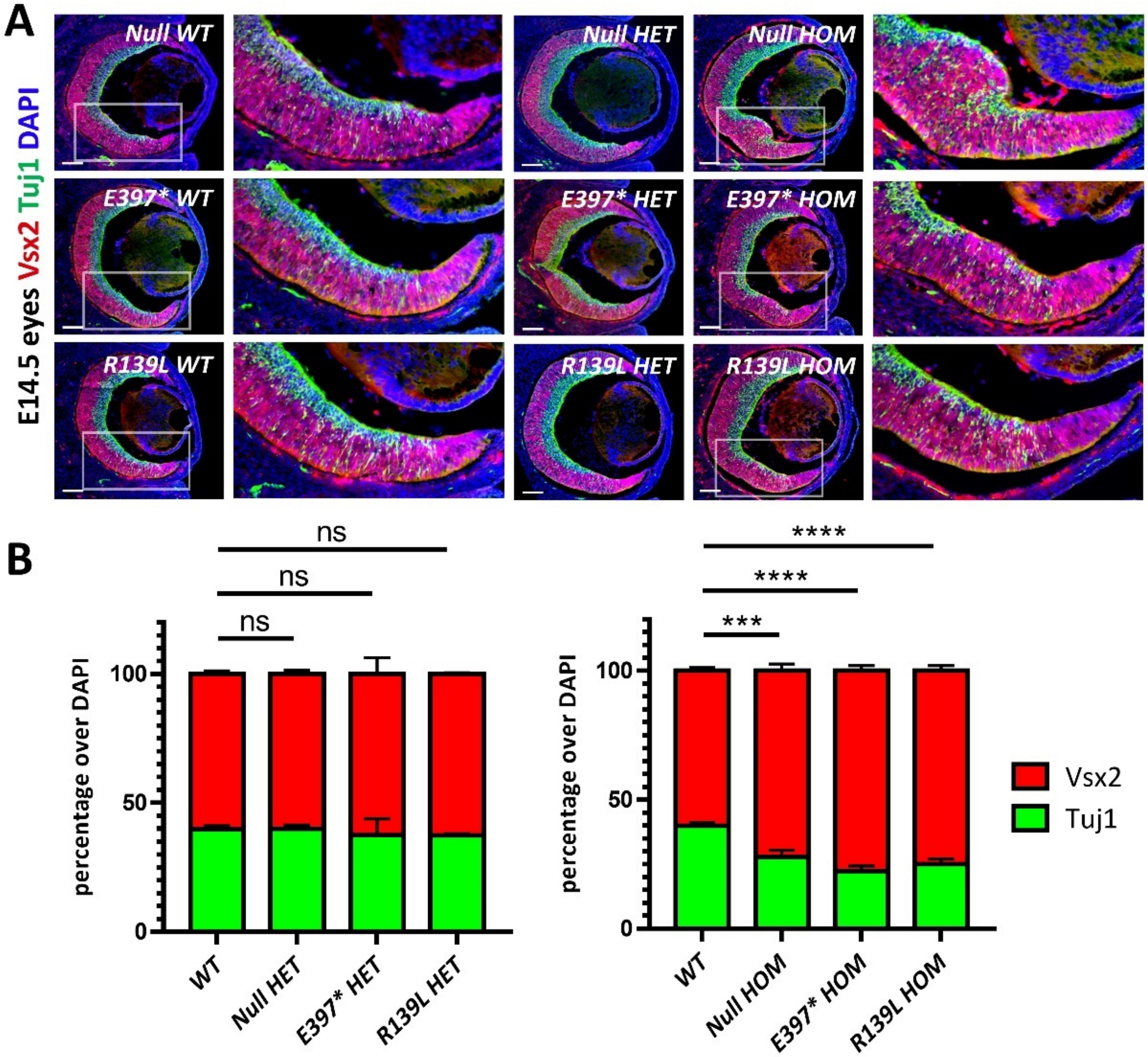

Fig. S2. ***Nr2f1*** **loss or mutation disrupts the balance between retinal progenitors and post-mitotic neurons. A)** Double immunostaining for Vsx2 (red; retinal progenitor cells) and Tuj1 (green; post-mitotic cells) in E14.5 retinae of the indicated genotypes. **B)** Quantification of the ratio of Vsx2$^+$ retinal progenitor cells to Tuj1$^+$ differentiated post-mitotic cells in the ventral retina of *WT* and mutant embryos, revealing a significant shift toward progenitor cells in *Null*, *E397** and *R139L HOM* mutants, consistent with delayed neurogenesis. Scale bars: 100 µm. For Graphs in B, n = 3-9 sections from n = 3-9 eyes per genotype. Statistical significance was determined by two-way ANOVA with Tukey's correction; *** $p < 0.001$; **** $p < 0.0001$.

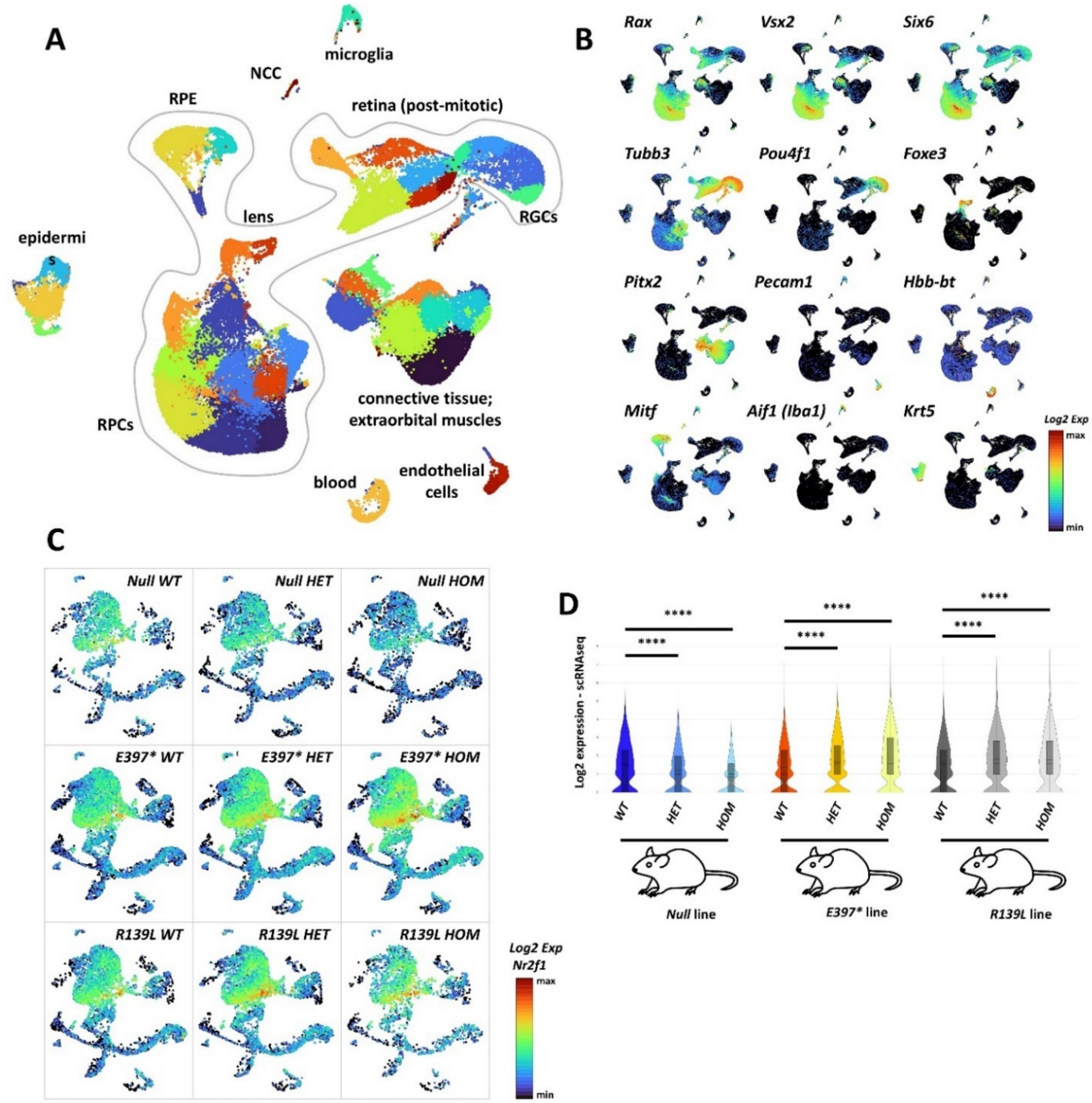

Fig. S3. Cell-type annotation and *Nr2f1* expression in E14.5 mouse retinae. A,B) UMAP projection of scRNA-seq data from E14.5 mouse eyes (A), showing distinct cell populations identified by *bona fide* tissue- and cell-type-specific markers (B). Retinal progenitor cells (RPCs) were identified by *Rax*$^{+}$, *Vsx2*$^{+}$, and *Six6*$^{+}$, whereas post-mitotic retinal populations, including retinal ganglion cells (RGCs), expressed *Tubb3* and *Pou4f1*. Retinal pigmented epithelium (RPE) was identified by *Foxe3* and *Mitf* expression. Additional non-retinal cell types arising from surrounding ocular tissues included *Krt5*$^{+}$ epidermal cells, *Aif1*$^{+}$ microglia, and *Pitx2*$^{+}$ connective tissue cells. **C,D)** *Nr2f1* mRNA expression levels in the re-clustered UMAP projection of E14.5 eye cells from the indicated genotypes (C), quantified using Loupe Browser (10x Genomics) and displayed as violin plots for each genotype (D). Statistical significance: **** $p < 0.0001$.

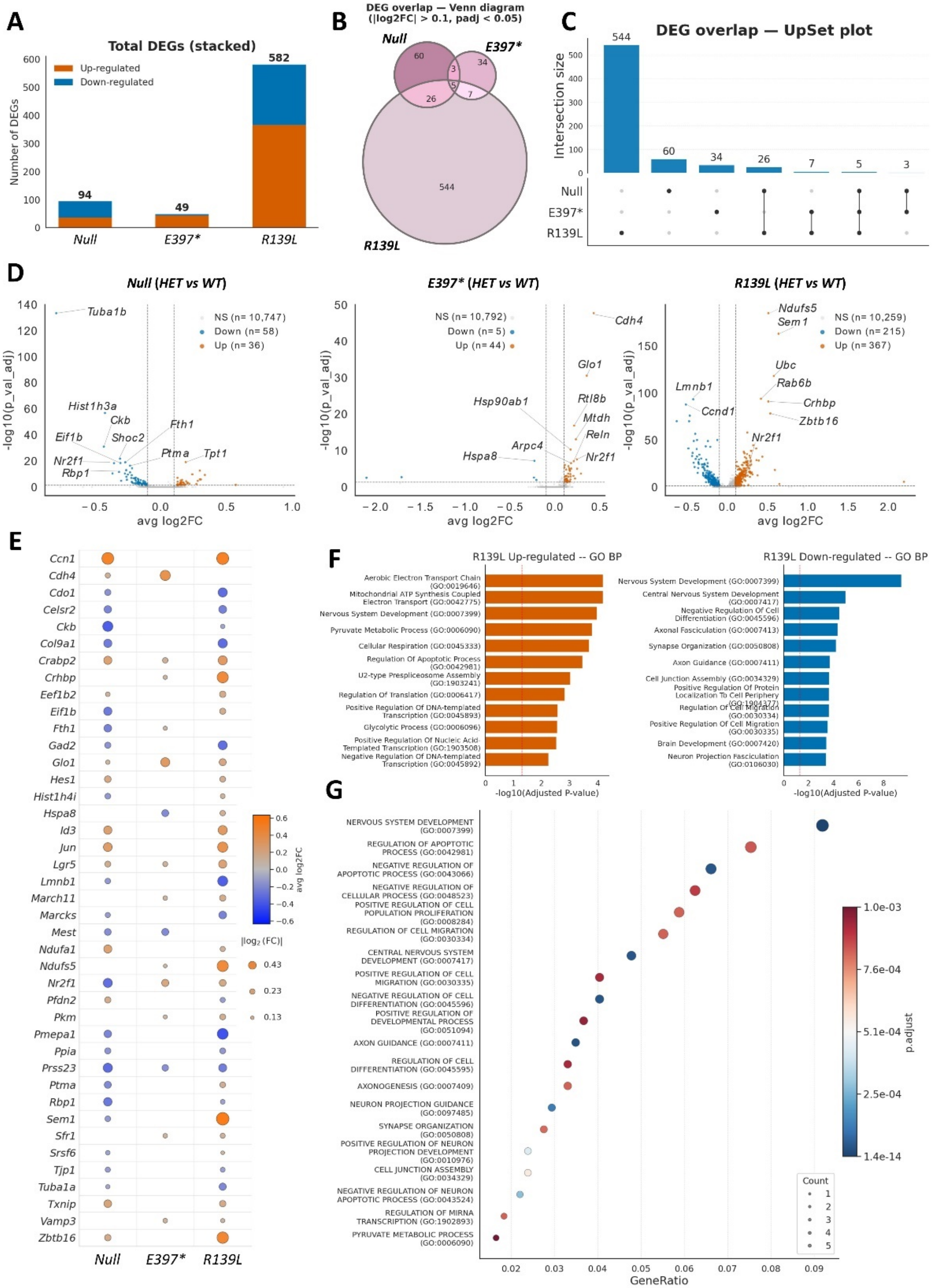

A
Total DEGs (stacked)
Up-regulated
Down-regulated
Number of DEGs
94
49
582
Null
E397*
R139L
B
DEG overlap — Venn diagram
(|log2FC| > 0.1, padj < 0.05)
Null
E397*
R139L
60
3
34
5
26
7
544
C
DEG overlap — UpSet plot
Intersection size
544
60
34
26
7
5
3
Null
E397*
R139L
D
Null (HET vs WT)
E397* (HET vs WT)
R139L (HET vs WT)
NS (n= 10,747)
Down (n= 58)
Up (n= 36)
NS (n= 10,792)
Down (n= 5)
Up (n= 44)
NS (n= 10,259)
Down (n= 215)
Up (n= 367)
-log10(p_val_adj)
avg log2FC
Tuba1b
Hist1h3a
Ckb
Eif1b
Shoc2
Fth1
Nr2f1
Ptma
Tpt1
Rbp1
Cdh4
Glo1
Hsp90ab1
Rtl8b
Mtdh
Reln
Arpc4
Hspa8
Nr2f1
Ndufs5
Sem1
Ubc
Rab6b
Lmnb1
Ccnd1
Crhbp
Zbtb16
Nr2f1
E
Ccn1
Cdh4
Cdo1
Celsr2
Ckb
Col9a1
Crabp2
Crhbp
Eef1b2
Eif1b
Fth1
Gad2
Glo1
Hes1
Hist1h4i
Hspa8
Id3
Jun
Lgr5
Lmnb1
March11
Marcks
Mest
Ndufa1
Ndufs5
Nr2f1
Pfdn2
Pkm
Pmepa1
Ppia
Prss23
Ptma
Rbp1
Sem1
Sfr1
Srsf6
Tjp1
Tuba1a
Txnip
Vamp3
Zbtb16
Null
E397*
R139L
avg log2FC
|log2 (FC)|
0.43
0.23
0.13
F
R139L Up-regulated -- GO BP
Aerobic Electron Transport Chain (GO:0019646)
Mitochondrial ATP Synthesis Coupled Electron Transport (GO:0042775)
Nervous System Development (GO:0007399)
Pyruvate Metabolic Process (GO:0006090)
Cellular Respiration (GO:0045333)
Regulation Of Apoptotic Process (GO:0042981)
U2-type Prespliceosome Assembly (GO:1903241)
Regulation Of Translation (GO:0006417)
Positive Regulation Of DNA-templated Transcription (GO:0045893)
Glycolytic Process (GO:0006096)
Positive Regulation Of Nucleic Acid-Templated Transcription (GO:1903508)
Negative Regulation Of DNA-templated Transcription (GO:0045892)
-log10(Adjusted P-value)
R139L Down-regulated -- GO BP
Nervous System Development (GO:0007399)
Central Nervous System Development (GO:0007417)
Negative Regulation Of Cell Differentiation (GO:0045596)
Axonal Fasciculation (GO:0007413)
Synapse Organization (GO:0050808)
Axon Guidance (GO:0007411)
Cell Junction Assembly (GO:0034329)
Positive Regulation Of Protein Localization To Cell Periphery (GO:1904377)
Regulation Of Cell Migration (GO:0030334)
Positive Regulation Of Cell Migration (GO:0030335)
Brain Development (GO:0007420)
Neuron Projection Fasciculation (GO:0106030)
-log10(Adjusted P-value)
G
NERVOUS SYSTEM DEVELOPMENT (GO:0007399)
REGULATION OF APOPTOTIC PROCESS (GO:0042981)
NEGATIVE REGULATION OF APOPTOTIC PROCESS (GO:0043066)
NEGATIVE REGULATION OF CELLULAR PROCESS (GO:0048523)
POSITIVE REGULATION OF CELL POPULATION PROLIFERATION (GO:0008284)
REGULATION OF CELL MIGRATION (GO:0030334)
CENTRAL NERVOUS SYSTEM DEVELOPMENT (GO:0007417)
POSITIVE REGULATION OF CELL MIGRATION (GO:0030335)
NEGATIVE REGULATION OF CELL DIFFERENTIATION (GO:0045596)
POSITIVE REGULATION OF DEVELOPMENTAL PROCESS (GO:0051094)
AXON GUIDANCE (GO:0007411)
REGULATION OF CELL DIFFERENTIATION (GO:0045595)
AXONOGENESIS (GO:0007409)
NEURON PROJECTION GUIDANCE (GO:0097485)
SYNAPSE ORGANIZATION (GO:0050808)
POSITIVE REGULATION OF NEURON PROJECTION DEVELOPMENT (GO:0010976)
CELL JUNCTION ASSEMBLY (GO:0034329)
NEGATIVE REGULATION OF NEURON APOPTOTIC PROCESS (GO:0043524)
REGULATION OF MIRNA TRANSCRIPTION (GO:1902893)
PYRUVATE METABOLIC PROCESS (GO:0006090)
GeneRatio
p.adjust
1.0e-03
7.6e-04
5.1e-04
2.5e-04
1.4e-14
Count

Fig. S4. Mutation-dependent transcriptional changes in *Nr2f1* heterozygous RPCs. A) Total number of up- (orange) and downregulated (blue) DEGs identified by comparing *WT* and *HET* retinal progenitor cells (RPCs) for each mouse line (*HET vs WT*). **B,C)** Venn diagram and intersection scheme illustrating the overlap of DEGs among the three mouse lines. Only a small number of genes are shared across all three models, whereas the *R139L* line displays several hundred DEGs. **D)** Volcano plots showing the magnitude of differential expression ($\log_2$ fold change; $\log_2$FC) and statistical significance ($-\log_{10}$ adjusted p value) for *WT vs HET* DEGs specific to each mouse line, as indicated. **E)** DEGs shared across two or three mouse lines, with upregulated and downregulated genes indicated in orange and blue, respectively. **F,G)** Gene ontology (GO) analysis of *R139L* DEGs, performed separately for up- and downregulated genes (F) or for the complete DEG set (G). A complete list of GO analyses for the other *WT vs HET* DEG subsets is provided as supplementary material.

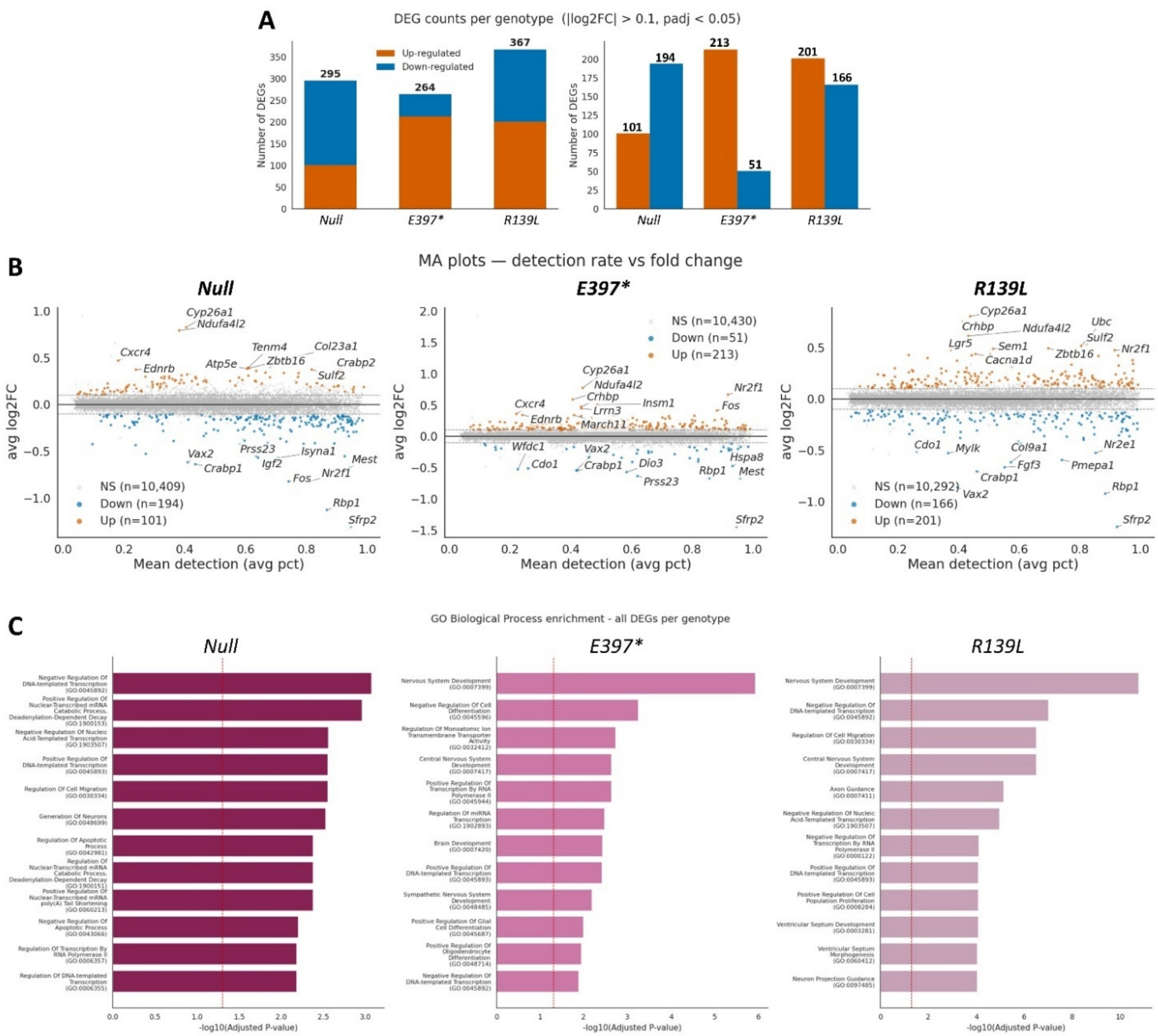

Fig. S5. Mutation-dependent transcriptional changes following complete *Nr2f1* loss or mutation. A) Number of up- (orange) and downregulated (blue) DEGs identified by comparing *WT* RPCs with their *KO/KI HOM* counterparts in the three mouse models (*WT vs HOM* DEGs). **B)** Plots showing up- (orange) and downregulated (blue) transcripts ranked according to the average proportion of cells expressing each gene (average percentage of cells; avg pct) across *WT* and mutant retinae for each mouse line, as indicated. **C)** Gene ontology analysis of all DEGs identified in each mouse model. A complete list of GO analyses for the other *WT vs HOM* DEG subsets is provided as supplementary material.

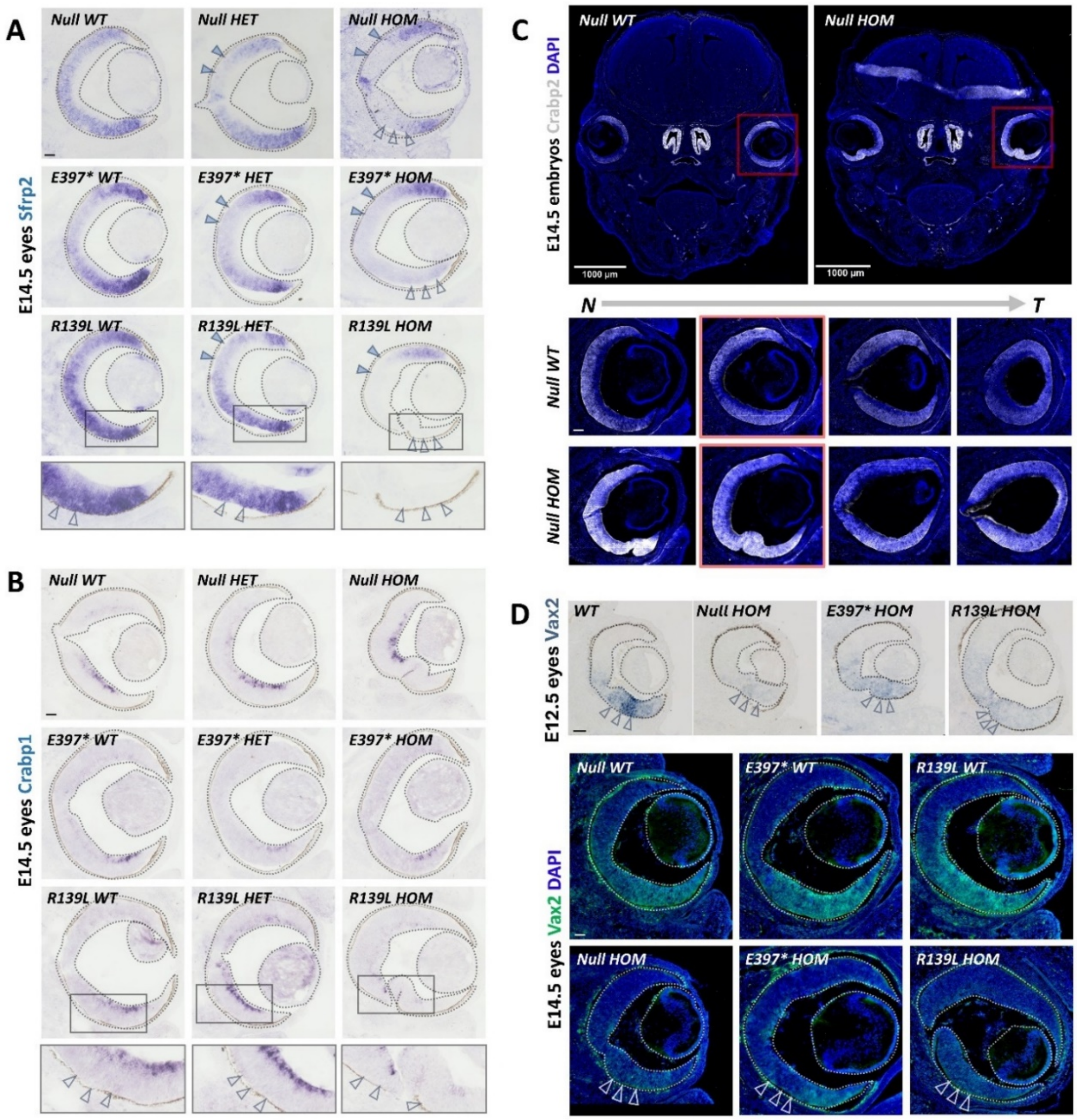

Fig. S6. Altered spatial expression of *Nr2f1*-dependent regional patterning genes in mutant retinae. A) *In situ* hybridization for *Sfrp2* transcript in E14.5 mouse retinae of the indicated genotypes. Blue arrowheads indicate reduced dorsal *Sfrp2* expression, already detectable in *HET* embryos, whereas white arrowheads indicate the ventral-most retina, where *Sfrp2* expression was completely lost specifically in *R139L HOM* mutants. **B)** *In situ* hybridization for *Crabp1* in E14.5 mouse retinae of the indicated genotypes. White arrowheads indicate reduced *Crabp1* expression in RPCs of *R139L* mutants, with a concomitant reduction in RGCs. **C)** Immunostaining for *Crabp2* (white) in E14.5 embryo heads, showing increased *Crabp2* expression in the ventral retina of *Null HOM* mutants at different positions along the naso (N)–temporal (T) axis. **D)** *In situ* hybridization for *Vax2* in E12.5 retinae and Vax2 immunostaining (green) in E14.5 retinae of the indicated genotypes, showing decreased expression in the ventral retina (arrowheads) of mutant embryos. Scale bars: 100 µm, except upper panel in C, 1000 µm.

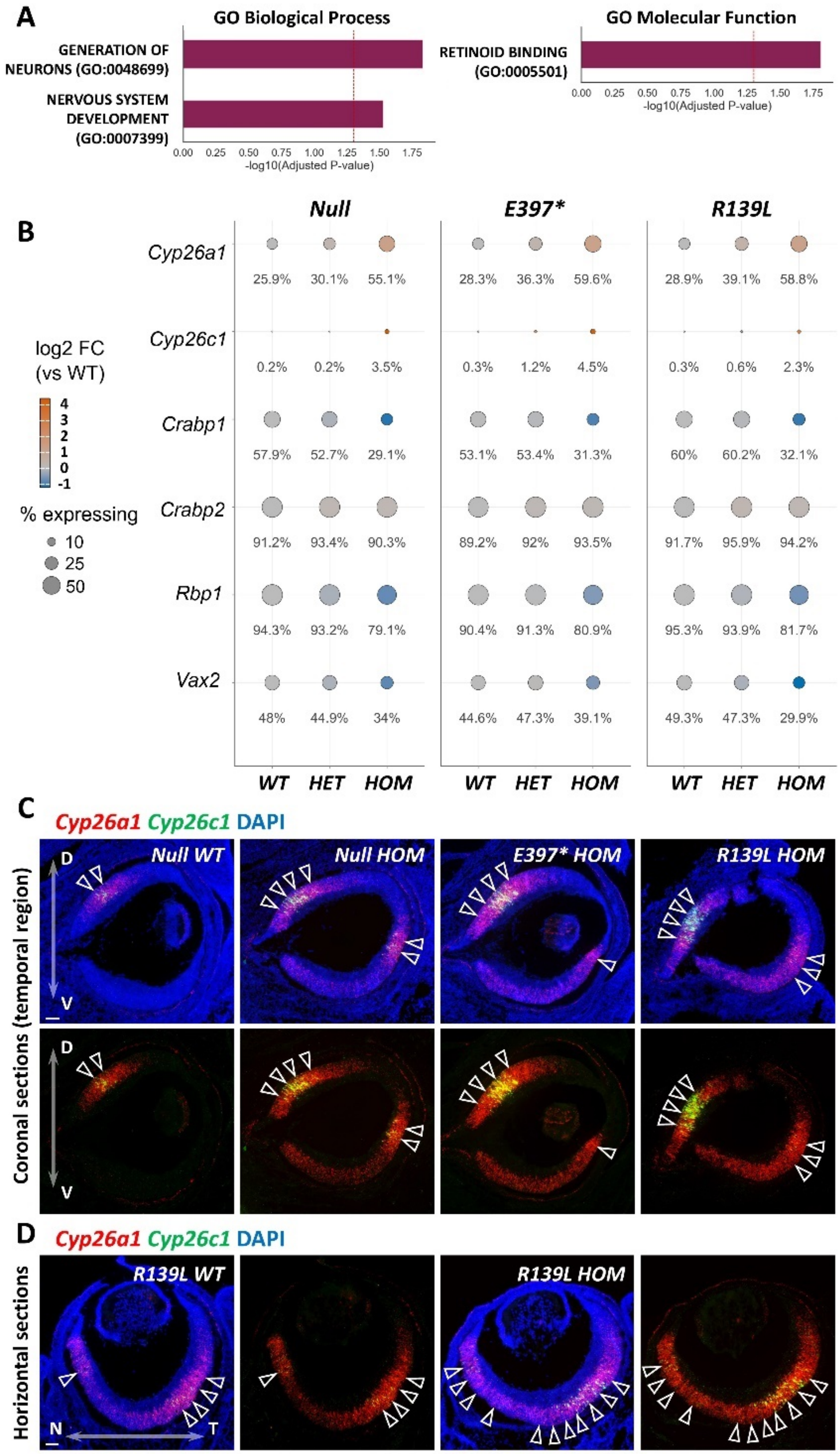
A
GO Biological Process
GENERATION OF NEURONS (GO:0048699)
NERVOUS SYSTEM DEVELOPMENT (GO:0007399)
-log10(Adjusted P-value)
GO Molecular Function
RETINOID BINDING (GO:0005501)
-log10(Adjusted P-value)
B
Null
E397*
R139L
Cyp26a1
Cyp26c1
Crabp1
Crabp2
Rbp1
Vax2
log2 FC (vs WT)
% expressing
WT HET HOM
C
Cyp26a1 Cyp26c1 DAPI
Coronal sections (temporal region)
Null WT
Null HOM
E397* HOM
R139L HOM
D
V
D
Cyp26a1 Cyp26c1 DAPI
Horizontal sections
R139L WT
R139L HOM
N
T

Fig. S7. Gene ontology and spatial analysis of *Nr2f1*-dependent RA pathway dysregulation. **A)** Gene ontology analysis of the 67 shared DEGs, highlighting "generation of neurons" and "nervous system development" as enriched GO biological processes, and "retinoid binding" as a significantly enriched molecular function. **B)** Dot plot showing the proportion of cells expressing the RA-related genes *Cyp26a1, Cyp26c1, Crabp1, Crabp2, Rbp1,* and *Vax2*, together with their fold change in *WT, HET* and *HOM KO/KI* animals for the indicated genotypes. **C)** RNAscope *in situ* hybridization for *Cyp26a1* (red) and *Cyp26c1* (green) in temporal-most coronal sections of E14.5 retinae from the indicated genotypes. Arrowheads indicate equato-dorsal retinal regions and ventral CMZ areas. Upregulation of both paralogs, together with ectopic *Cyp26a1*expression, is particularly pronounced in the ventral retina (D = dorsal, V = ventral). **D)** RNAscope *in situ* hybridization for *Cyp26a1* (red) and *Cyp26c1* (green) in horizontal sections of E14.5 *WT* and *R139L HOM KI* retinae. Arrowheads point to high Cyp26a1 expression. Ectopic upregulation of Cyp26a1 is detected in the nasal retina (N = nasal, T = temporal). Scale bars: 50 µm.

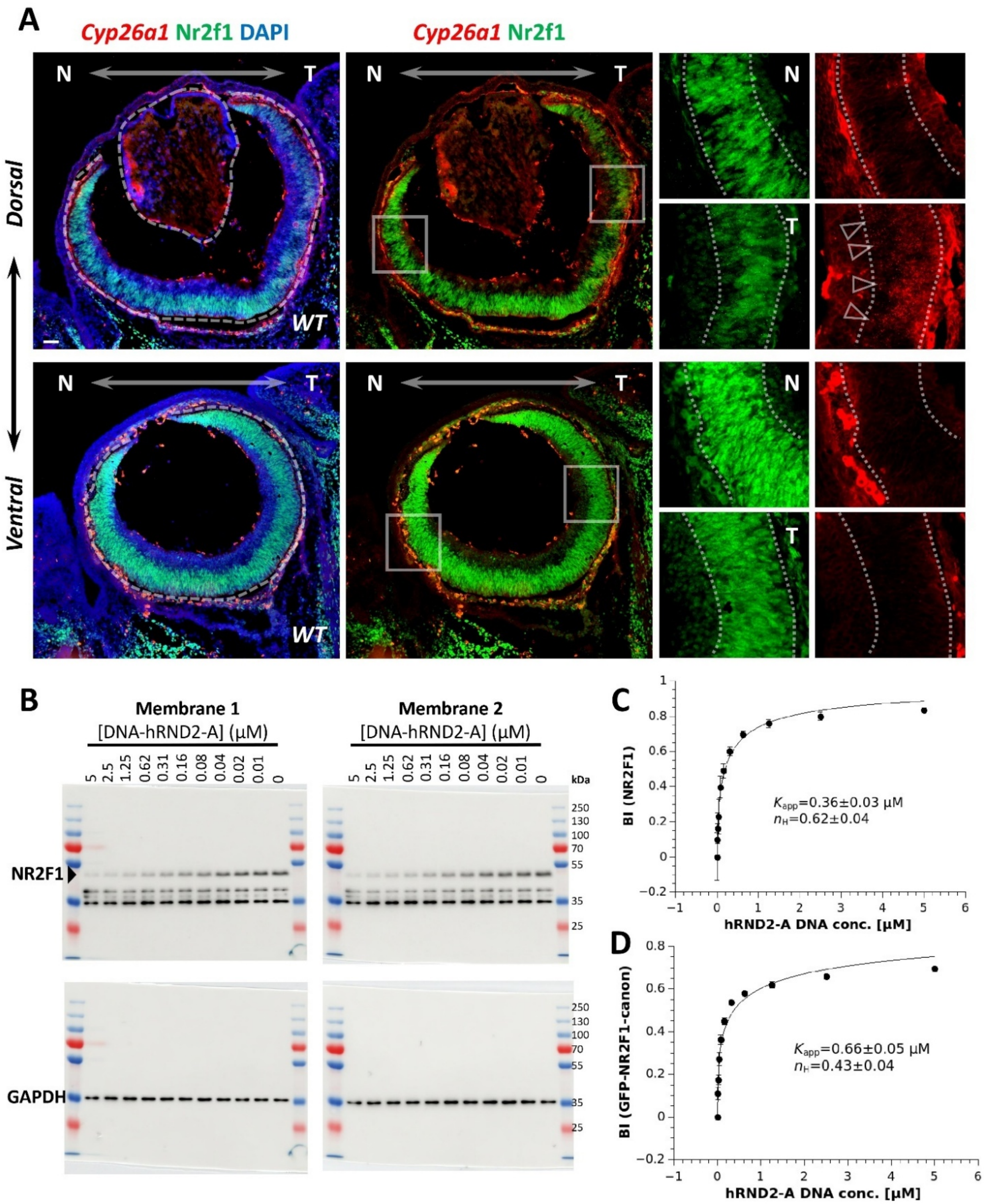
A
Cyp26a1 Nr2f1 DAPI
Cyp26a1 Nr2f1
N
T
Dorsal
Ventral
WT
B
Membrane 1
[DNA-hRND2-A] (μM)
5 2.5 1.25 0.62 0.31 0.16 0.08 0.04 0.02 0.01 0
Membrane 2
[DNA-hRND2-A] (μM)
5 2.5 1.25 0.62 0.31 0.16 0.08 0.04 0.02 0.01 0
kDa
250 130 100 70 55 35 25
NR2F1
GAPDH
C
BI (NR2F1)
K_{app}=0.36±0.03 μM
n_H=0.62±0.04
hRND2-A DNA conc. [μM]
D
BI (GFP-NR2F1-canon)
K_{app}=0.66±0.05 μM
n_H=0.43±0.04
hRND2-A DNA conc. [μM]

Fig. S8. Complementary N-T expression gradients of Nr2f1 and *Cyp26a1* and validation of NR2F1 DNA-binding activity by native holdup. A) RNAscope *in situ* hybridization for *Cyp26a1* (red) combined with Nr2f1 immunostaining (green) on E14.5 WT eye horizontal sections at the indicated dorsal and ventral levels. Nr2f1 exhibits a nasal (N)-to-temporal (T) decreasing gradient, more pronounced at the dorsal level. Conversely, sparse *Cyp26a1* expression (arrowheads in inset) is detected predominantly in the temporal retina, where Nr2f1 expression levels are lower. **B–D)** Native holdup analysis using endogenous NR2F1 from neural progenitor cells and GFP-tagged NR2F1 expressed in HEK293T cells. An NR2F1 recognition sequence from the human *RND2* gene was used as DNA bait. NR2F1 prey proteins were captured directly from cell extracts and detected by Western blotting or GFP fluorescence. In B, Western blot analysis of native holdup performed with endogenous NR2F1 from neural progenitor stem cell (NPSC) extracts (day 12 of differentiation). C shows the binding curve derived from the Western blot quantification shown in B (n = 2). In D**,** the binding curve obtained from native holdup using GFP-NR2F1 expressed in HEK293T cells (n = 3). BI, bound fraction intensity; Kapp, apparent affinity; nH, Hill coefficient. Scale bars: 50 µm.

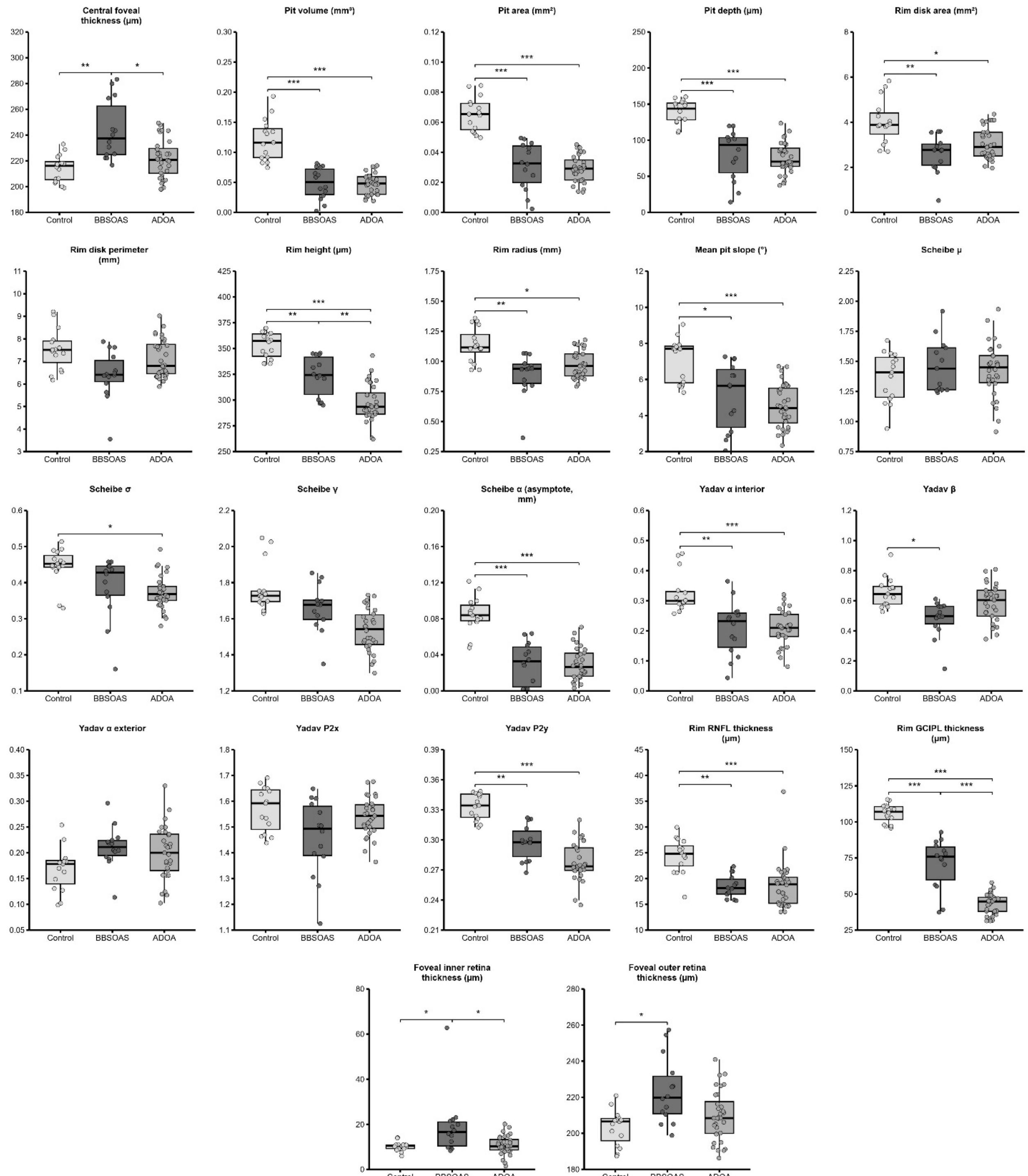
Central foveal thickness (µm)
Pit volume (mm³)
Pit area (mm²)
Pit depth (µm)
Rim disk area (mm²)
Rim disk perimeter (mm)
Rim height (µm)
Rim radius (mm)
Mean pit slope (°)
Scheibe µ
Scheibe σ
Scheibe γ
Scheibe α (asymptote, mm)
Yadav α interior
Yadav β
Yadav α exterior
Yadav P2x
Yadav P2y
Rim RNFL thickness (µm)
Rim GCIPL thickness (µm)
Foveal inner retina thickness (µm)
Foveal outer retina thickness (µm)
Control
BBSOAS
ADOA

Fig. S9. Comparison of foveal morphology across BBSOAS, ADOA, and healthy controls. Boxplots comparing 22 quantitative foveal pit morphology parameters among individuals with BBSOAS (n = 14 eyes from 7 patients; dark grey), autosomal dominant optic atrophy (ADOA; n = 32 eyes from 16 patients; grey), and age-matched healthy controls (n = 16 eyes from 8 individuals; light grey). Boxes represent the interquartile range, with the median indicated. Statistical significance was assessed using linear mixed-effects models accounting for measurements from both eyes, with P values adjusted for multiple comparisons using the Benjamini–Hochberg procedure (*$P < 0.05$, **$P < 0.01$, ***$P < 0.001$). Parameters were grouped into four categories: (i) foveal pit morphology derived from radial retinal thickness profiles (central foveal thickness, pit volume/area/depth, rim disk area/perimeter, rim height/radius, mean pit slope); (ii) Scheibe et al. radial parametric fit coefficients (μ, σ, γ, α); (iii) Yadav et al. cubic-Bézier fit coefficients (α interior/exterior, β, P2x, P2y); and (iv) retinal layer thicknesses measured at the pit rim (RNFL, GCIPL) and foveal center (inner retina = ILM to IPL/INL; outer retina = IPL/INL to BM). Demographic characteristics of patients with BBSOAS, patients with ADOA, and control individuals included in the analysis are provided in *Table S1*; *NR2F1* and *OPA1* pathogenic variants in the patients included in this analysis are listed in *Tables S2* and *S4*, respectively. Summary statistics for the foveal parameters shown in the graphs are provided in *Table S5*.

Cohort	n_patients	n_eyes	n_female	pct_female	age_mean	age_sd	age_range	summary
Control	8	16	4	50	281	5	23-37	n=8 (16 eyes), 50% female, age at scan 28.1 +/- 5.0 (23-37)
ADOA	16	32	8	50	403	94	23-57	n=16 (32 eyes), 50% female, age at scan 40.3 +/- 9.4 (23-57)
BBSOAS	7	14	4	57	361	209	16-75	n=7 (14 eyes), 57% female, age at scan 36.1 +/- 20.9 (16-75)

Table S1. Demographic summary of control, ADOA and BBSOAS individuals.

BBSOAS_patient_genotype_list

Study ID	Gene	cDNA (HGVS)	Protein	Family	In original paper
**BB-01**	NR2F1	c.1115T>C	p.(Leu372Pro)	F1	Yes
**BB-02**	NR2F1	c.1115T>C	p.(Leu372Pro)	F1	Yes
**BB-03**	NR2F1	c.1115T>C	p.(Leu372Pro)	F1	Yes
**BB-04**	NR2F1	c.1118_1123del	p.(Arg373_Leu374del)	F2	Yes
**BB-05**	NR2F1	c.1036_1047del	p.(Glu346_Gln349del)	F3	Yes
**BB-06**	NR2F1	c.237G>C	p.(Gln79His)	F4	No
**BB-07**	NR2F1	(not recorded)	(not recorded)	F5	No

Table S2. *NR2F1* pathogenic variants in individuals with BBSOAS included in the foveal morphometric analysis

BBSOAS_vs_Control_summary_table

Metric	Control (8 patients, 16 eyes)	BBSOAS (7 patients, 14 eyes)	p (BH-adj)
Central foveal thickness (μm)	214.16 ± 10.33	243.37 ± 22.98	0.015
Foveal inner retina thickness (μm)	10.23 ± 2.10	18.94 ± 13.62	0.065
Foveal outer retina thickness (μm)	203.32 ± 9.86	223.42 ± 18.41	0.025
Mean pit slope (°)	7.12 ± 1.20	5.07 ± 1.85	0.039
Pit area (mm²)	0.07 ± 0.01	0.03 ± 0.02	0.002
Pit depth (μm)	139.18 ± 16.11	79.94 ± 34.64	0.003
Pit volume (mm³)	0.12 ± 0.03	0.05 ± 0.03	0.003
Rim GCIPL thickness (μm)	105.78 ± 6.40	70.91 ± 17.41	0.002
Rim RNFL thickness (μm)	24.46 ± 3.33	18.44 ± 2.19	0.002
Rim disk area (mm²)	4.03 ± 0.97	2.60 ± 0.83	0.015
Rim disk perimeter (mm)	7.53 ± 0.91	6.40 ± 1.10	0.044
Rim height (μm)	353.34 ± 12.64	323.31 ± 19.07	0.010
Rim radius (mm)	1.14 ± 0.14	0.89 ± 0.18	0.015
Scheibe α (asymptote, mm)	0.08 ± 0.02	0.03 ± 0.02	0.002
Scheibe γ	1.77 ± 0.13	2.80 ± 4.27	0.358
Scheibe μ	1.37 ± 0.21	2.57 ± 4.15	0.308
Scheibe σ	0.45 ± 0.05	0.39 ± 0.09	0.150
Yadav P2x	1.57 ± 0.09	1.46 ± 0.15	0.069
Yadav P2y	0.33 ± 0.01	0.30 ± 0.02	0.003
Yadav α exterior	0.17 ± 0.04	0.21 ± 0.04	0.044
Yadav α interior	0.32 ± 0.07	0.21 ± 0.09	0.015
Yadav β	0.66 ± 0.10	0.48 ± 0.12	0.014

Table S3. Summary table of the foveal morphological parameters measured for both control and individuals with BBSOAS.

ADOA_patient_genotype_list

Study ID	Gene	cDNA (HGVS)	Family
**ADOA-01**	OPA1	c.1241C>G	F1
**ADOA-02**	OPA1	c.1241C>G	F1
**ADOA-03**	OPA1	c.1149G>A	
**ADOA-04**	OPA1	c.2708_2711del	
**ADOA-05**	OPA1	c.869G>A	
**ADOA-06**	OPA1	c.2818+1G>A	
**ADOA-07**	OPA1	c.2521-2A>G	
**ADOA-08**	OPA1	c.2873_2876delTTAG	
**ADOA-09**	OPA1	c.2706_2711del	
**ADOA-10**	OPA1	c.1140G>A	
**ADOA-11**	OPA1	c.870+2T>A	
**ADOA-12**	OPA1	Deletion of exons 2–4 (~2.6 kb)	
**ADOA-13**	OPA1	c.1212+1G>A	
**ADOA-14**	OPA1	c.1491_1493delins37	
**ADOA-15**	OPA1	c.899G>A	
**ADOA-16**	OPA1	c.1187T>G	

Table S4. *OPA1* pathogenic variants in individuals with ADOA included in the foveal morphometric analysis.

Control_BBSOAS_ADOA_summary_table

Metric	Control (8 patients, 16 eyes)	BBSOAS (7 patients, 14 eyes)	ADOA (16 patients, 32 eyes)	Omnibus p (BH-adj)	BBSOAS vs Control (Tukey)	ADOA vs Control (Tukey)	BBSOAS vs ADOA (Tukey)
**Central foveal thickness (µm)**	214.16 ± 10.33	243.37 ± 22.98	221.56 ± 15.03	0.008	0.005	0.554	0.017
**Pit volume (mm^3)**	0.12 ± 0.03	0.05 ± 0.03	0.05 ± 0.02	<0.001	<0.001	<0.001	0.997
**Pit area (mm^2)**	0.07 ± 0.01	0.03 ± 0.02	0.03 ± 0.01	<0.001	<0.001	<0.001	0.933
**Pit depth (µm)**	139.18 ± 16.11	79.94 ± 34.64	75.66 ± 21.18	<0.001	<0.001	<0.001	0.919
**Rim disk area (mm^2)**	4.03 ± 0.97	2.60 ± 0.83	3.04 ± 0.70	0.005	0.003	0.012	0.453
**Rim disk perimeter (mm)**	7.53 ± 0.91	6.40 ± 1.10	7.13 ± 0.84	0.056	0.037	0.472	0.173
**Rim height (µm)**	353.34 ± 12.64	323.31 ± 19.07	297.22 ± 18.03	<0.001	0.007	<0.001	0.007
**Rim radius (mm)**	1.14 ± 0.14	0.89 ± 0.18	0.98 ± 0.11	0.006	0.003	0.022	0.353
**Mean pit slope (°)**	7.12 ± 1.20	5.07 ± 1.85	4.52 ± 1.20	0.002	0.022	<0.001	0.662
**Scheibe µ**	1.37 ± 0.21	2.57 ± 4.15	1.43 ± 0.23	0.198	0.273	0.995	0.217
**Scheibe σ**	0.45 ± 0.05	0.39 ± 0.09	0.37 ± 0.05	0.036	0.162	0.022	0.826
**Scheibe γ**	1.77 ± 0.13	2.80 ± 4.27	1.54 ± 0.12	0.178	0.384	0.932	0.160
**Scheibe α (asymptote, mm)**	0.08 ± 0.02	0.03 ± 0.02	0.03 ± 0.02	<0.001	<0.001	<0.001	0.999
**Yadav α interior**	0.32 ± 0.07	0.21 ± 0.09	0.21 ± 0.06	0.001	0.004	<0.001	0.997
**Yadav β**	0.66 ± 0.10	0.48 ± 0.12	0.58 ± 0.12	0.022	0.011	0.308	0.107
**Yadav α exterior**	0.17 ± 0.04	0.21 ± 0.04	0.20 ± 0.05	0.112	0.112	0.181	0.820
**Yadav P2x**	1.57 ± 0.09	1.46 ± 0.15	1.54 ± 0.08	0.056	0.046	0.682	0.120
**Yadav P2y**	0.33 ± 0.01	0.30 ± 0.02	0.28 ± 0.02	<0.001	0.002	<0.001	0.057
**Rim RNFL thickness (µm)**	24.46 ± 3.33	18.44 ± 2.19	18.71 ± 4.43	<0.001	0.002	<0.001	0.978
**Rim GCIPL thickness (µm)**	105.78 ± 6.40	70.91 ± 17.41	42.95 ± 7.18	<0.001	<0.001	<0.001	<0.001
**Foveal inner retina thickness (µm)**	10.23 ± 2.10	18.94 ± 13.62	10.68 ± 4.19	0.019	0.028	0.986	0.015
**Foveal outer retina thickness (µm)**	203.32 ± 9.86	223.42 ± 18.41	209.68 ± 13.87	0.033	0.020	0.477	0.097

Table S5. Summary table of the foveal morphological parameters measured for control, ADOA and BBSOAS individuals.
